# Ventricle stress/strain comparisons between Tetralogy of Fallot patients and healthy using models with different zero-load diastole and systole morphologies

**DOI:** 10.1371/journal.pone.0220328

**Published:** 2019-08-14

**Authors:** Han Yu, Dalin Tang, Tal Geva, Chun Yang, Zheyang Wu, Rahul H. Rathod, Xueying Huang, Kristen L. Billiar, Pedro J. del Nido

**Affiliations:** 1 School of Biological Science & Medical Engineering, Southeast University, Nanjing, China; 2 Mathematical Sciences Department, Worcester Polytechnic Institute, Worcester, MA, United States of America; 3 Department of Cardiology, Boston Children's Hospital, Department of Pediatrics, Harvard Medical School, Boston, MA, United States of America; 4 School of Mathematical Sciences, Xiamen University, Xiamen, Fujian, China; 5 Department of Biomedical Engineering, Worcester Polytechnic Institute, Worcester, MA, United States of America; 6 Department of Cardiac Surgery, Boston Children’s Hospital, Department of Surgery, Harvard Medical School, Boston, MA, United States of America; Freeman Hospital, UNITED KINGDOM

## Abstract

Patient-specific in vivo ventricle mechanical wall stress and strain conditions are important for cardiovascular investigations and should be calculated from correct zero-load ventricle morphologies. Cardiac magnetic resonance (CMR) data were obtained from 6 healthy volunteers and 12 Tetralogy of Fallot (TOF) patients with consent obtained. 3D patient-specific CMR-based ventricle models with different zero-load diastole and systole geometries due to myocardium contraction and relaxation were constructed to qualify right ventricle (RV) diastole and systole stress and strain values at begin-filling, end-filling, begin-ejection, and end-ejection, respectively. Our new models (called 2G models) can provide end-diastole and end-systole stress/strain values which models with one zero-load geometries (called 1G models) could not provide. 2G mean end-ejection stress value from the 18 participants was 321.4% higher than that from 1G models (p = 0.0002). 2G mean strain values was 230% higher than that of 1G models (p = 0.0002). TOF group (TG) end-ejection mean stress value was 105.4% higher than that of healthy group (HG) (17.54±7.42kPa vs. 8.54±0.92kPa, p = 0.0245). Worse outcome group (WG, n = 6) post pulmonary valve replacement (PVR) begin-ejection mean stress was 57.4% higher than that of better outcome group (BG, 86.94±26.29 vs. 52.93±22.86 kPa; p = 0.041). Among 7 selected parameters, End-filling stress was the best predictor to differentiate BG patients from WG patients with prediction accuracy = 0.8208 and area under receiver operating characteristic curve (AUC) value at 0.8135 (EE stress). Large scale studies are needed to further validate our findings.

## Introduction

Patients with repaired tetralogy of Fallot (TOF) account for the majority of cases with late onset right ventricular (RV) failure. Surgical repair of TOF usually involves incision into the right ventricular outflow tract (RVOT), pulmonary valve replacement/insertion (PVR), removal of obstructive cardiac muscle, removal of obstructive pulmonary valve tissue, and placement of a patch made of non-contracting tissue or synthetic material to widen the RVOT and pulmonary valve annulus. The current surgical approach, which includes PVR and often RV remodeling, has yielded mixed results with many patients not recovering RV function after pulmonary valve insertion with or without concomitant RV remodeling surgery [1,2,3]. Therefore, there is a need to identify potential predictors which could identify patients with better post-PVR outcome from patient with worse post-PVR outcome [4,5].

It is well accepted that mechanical stress and strain conditions play an important role in cardiovascular disease initiation, development, treatment, organ remodeling, and surgical recovery process. Accurate ventricle stress and strain calculations are of fundamental importance for ventricle disease assessment, surgical design, and post-surgical recovery. McCulloch et al., Hunter et al., Holmes et al., Costa et al., and many other authors have made great contributions to passive and active ventricle modeling, including the Physiome Project and the Continuity package [6–11]. Early cardiac magnetic resonance imaging (CMR)-based ventricle models were introduced by Axel and Saber et al. for mechanical analysis and investigations [11, 12]. We have introduced patient-specific CMR image-based computational right and left ventricle (RV/LV) models with fluid-structure interactions (FSI) to assess outcomes of various RV reconstruction techniques with different scar tissue trimming and patch sizes [4, 5, 13, 14, 15]. A recent review can be found in [15].

From mechanical point of view, zero-stress ventricle geometry information is required for its stress/strain calculations. Computational models often need to start from geometries with zero stress/strain conditions. Due to active contraction and myocardium sarcomere zero-stress length shortening, ventricle zero-load geometries for diastole and systole phases are different, with zero-load systole geometry being smaller than that of diastole phase. In this paper, different zero-load systole and diastole ventricle geometries were used to obtain more accurate stress/strain calculations: one zero-load ventricle geometry is used to model the diastole phase where sarcomere has its relaxed zero-stress length; another zero-load ventricle geometry is used to model the systole phase where sarcomere has its contracted zero-stress length. Essentially, we are using two models (called 2G models) to model the cardiac cycle to handle the active contraction and relaxation which are caused by zero-stress sarcomere length changes. It should be noted that zero-stress and zero-load are two different concepts. Zero-load geometries are used as an approximation since zero-stress state is really hard to get, and zero-load geometries are what we need for model construction purposes.

In this paper, patient-specific 2G models were constructed for 6 healthy volunteers and 12 TOG patients based on cardiac magnetic resonance (CMR) data to obtain ventricle stress/strain conditions. Results from 2G models were compared with that from previous models with one zero-load geometry to observe the improvements by the 2G models. Differences between systole and diastole stress/strain conditions and between healthy and TOG patients were identified. Ventricle stress/strain and other morphological parameters were used to differentiate patients with better post-PVR outcome from patients with worse post-PVR outcome to identify potential possible predictors. Ventricle stress and strain of healthy and TOG patients calculated using different zero-load diastole and systole morphologies will be good contributions to the literature.

## Methods

### 2.1 Data acquisition and 3D geometry reconstruction

**Fig 1:**
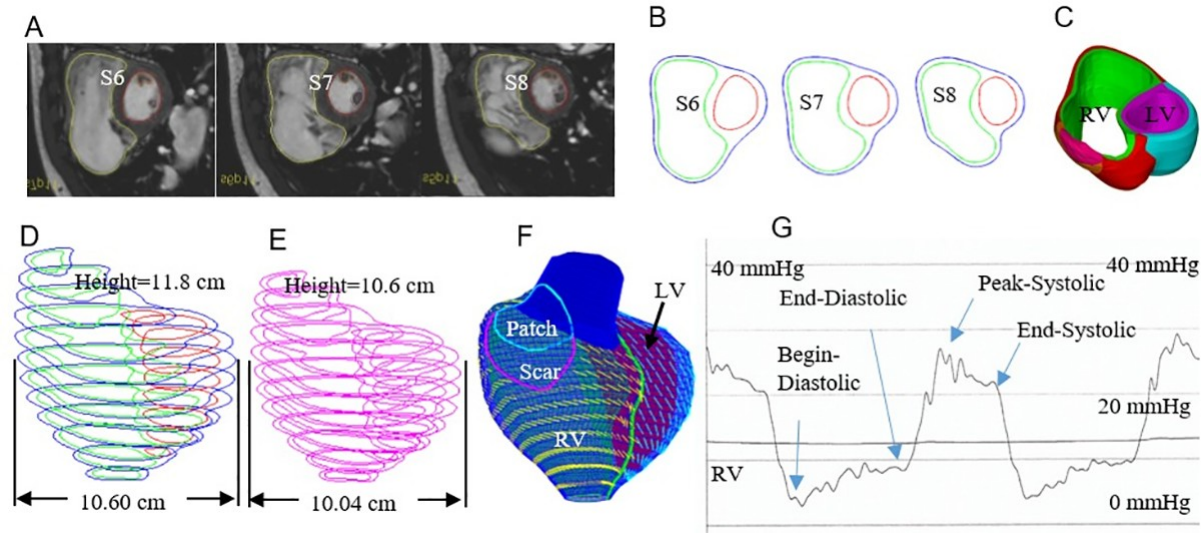
Illustration of CMR–based model construction process and pressure conditions. (A) Selected CMR slices from a patient, begin of diastole. (B) Segmented contours. (C) Two-layer myocardium structure. (D) Zero-load diastole geometry. (E) Zero-load systole geometry. (F) Model with fiber orientations. (G) Recorded RV pressure profile.

The study was approved by Boston Children’s Hospital Committee on Clinical Investigation. The IRB approval number is: IRB-CRM09-04-0237. Written informed consent was obtained from participants. CMR data from 6 healthy volunteers and 12 TOF patients before and 6 months after PVR were obtained. Demographic and CMR data for the 18 participants (11 male, average age 32.1) are given by Table 1. The 18 participants were divided into healthy group (HG) and TOF group (TOG) for comparison purposes. Depending on the post-PVR outcome, 12 TOF patients were further divided into Better-outcome group (BG) and Worse-outcome group (WG) corresponding to the post-PVR outcome for prediction analysis. Ventricle ejection fraction (EF) is commonly used as a measure for ventricle cardiac functions. In this paper, EF variance after PVR (ΔEF, difference between post-PVR EF and pre-PVR EF) was used as the criterion for BG and WG. Since we only have a small group, for the 12 TOF patients, the “best” way for us is to divide them into two even groups, 6 in BG and 6 in WG. This is purely for better comparisons with no clinical reasons. Ideally, we should use positive and negative ΔEF as a criterion for BG and WG. That would give us 4 in BG and 8 in WG, leading to poorer results and uneven comparisons. For our even division (BG = 6, WG = 6), the median ΔEF (-5%) was used as our criterion, i.e., TOF patients with ΔEF>-5% were classified into better group, and the rest were classified into worse group. P-values from Wilcoxon rank sum test in Table 1 indicated that there was no significant age difference between BG and WG, and between HG and TG (TOF patient group). Data acquisition techniques were previously published [14]. Image segmentation were performed with commercial software (QMass, Medis Medical Imaging Systems, Leiden, the Netherlands). Simpson’s method was used to calculate end-diastolic volume (EDV) and end-systolic volume (ESV). Stroke volume and ejection fraction were determined from EDV and ESV. Location of patch and valve of TOF patients were determined with flow data, cine MR imaging, and delayed enhancement CMR, and were further verified by the surgeon (PJdN, over 30 years of experience) who performed PVR for those patients. Ventricle pressure data from TOF patients were obtained from cardiac catheterization procedures. RV pressure in healthy controls was derived from Doppler measurement of the tricuspid regurgitation jet velocity by echocardiography. Fig 1 shows 3 selected CMR slices, corresponding segmented contour plots, zero-load systole and diastole ventricle geometries, reconstructed 3D RV/LV geometry with scar, patch and myocardium fiber orientation, and measured ventricle diastole and systole pressure conditions from a TOF patient.

**Table 1:** Demographic and CMR data for healthy human and TOF patients.

Patient no.	Sex	Age (y)	Maximum Pressure (mmHg)	RV EDV (cm^3^)	RVEDVi(ml/m^2^)	RV ESV (cm^3^)	EVESVi(ml/m^2^)	RV EF (%)	ΔEF (%)
**Healthy Group**									
P1	F	46.7	22	128.4		46.9		63	-
P2	M	23.6	27.9	226.6		105.4		53	-
P3	M	20.8	24	231.7		107.0		54	-
P4	M	19.4	23.8	213.5		94.2		56	-
P5	M	17.7	24.3	233.7		105.5		55	-
P6	M	6.7	24.8	67.6		28.2		58	-
**HG Mean**		22.5	24.5	183.6		81.2		56.5	-
**± SD**		±13.2	±1.93	±69.4		±34.6		±3.6	
**Better-outcome group**									
P7	M	47.7	31	408.8	192.68	254.8	120.10	37.7	-2.6
P8	M	50.0	33	364.6	205.10	239.5	134.75	34.3	-2.9
P9	F	42.0	45	323.3	178.60	177.8	98.24	45.0	4.0
P10	F	14.3	29	204.0	175.86	104.3	89.91	48.8	5.6
P11	F	15.3	15	193.7	186.25	105.1	101.06	45.7	6.6
P12	M	17.0	27	188.3	229.63	108.3	132.07	42.5	2.0
**BG Mean**		31.1	30.0	280.5	194.7	165.0	112.7	42.3	2.1
**± SD**		±17.2	±9.7	±97.2	±20.1	±69.7	±18.9	±5.4	±4.1
**Worse-outcome group**									
P13	F	56.9	41	385.1	224.73	184.6	106.80	52.1	-18.0
P14	M	11.6	36	204.2	167.35	121.3	99.39	40.6	-8.4
P15	M	43.5	65	665.1	263.99	464.0	184.20	30.2	-15.2
P16	M	54.1	63	334.8	155.38	170.8	79.25	49.0	-7.0
P17	F	44.6	50	299.0	184.57	186.0	114.81	37.8	-12.3
P18	F	45.3	49	571.1	291.38	371.3	189.44	35.0	-13.4
**WG Mean**		42.7	50.7	409.9	214.6	249.7	129.0	40.8	-12.
**± SD**		±16.2	±11.6	±174.3	±54.9	±135.4	±46.4	±8.4	±4.1
**BG, WG p-value**		0.394	0.009	0.180	0.937	0.240	0.818	0.937	0.002
**TOF mean**		36.9	40.	345.2	204.6	207.3	120.8	41.6	-5.1
**± SD**		±17.0	±14.8	±150.6	±40.8	±111.8	±34.8	±6.8	±8.5
**HG, TOG p-value**		0.291	0.005	0.067	-	0.003	-	0.000	-
**HG, TOF Mean**		32.1	35.0	291.3		165.3		46.5	
**± SD**		±17.0	±14.2	±149.0		±110.4		±9.3	

### 2.2 Stress/strain definition and calculations and the pre-shrink process to obtain zero-load diastole and systole geometries

**Fig 2:**
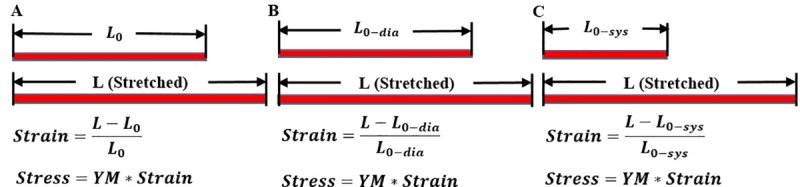
Stress and strain calculation and illustratiom of the concept of zero-stress myocardium sarcomere length in diastole and systole. A. A zero-stress bar with length L_0_ and its stretched length L to illustrate stress/strain definition. B. illustration of zero-stress myocardium sarcomere length and its in vivo stressed length in diastole; C. illustration of shortened zero-stress myocardium sarcomere length and its in vivo stressed length in systole. YM: Young’s Modulus.

Mechanical stress and strain are defined and should be calculated using zero-stress reference frames. Fig 2 gave an illustration of the concept using a one-dimensional bar. Fig 2 (A) gives a bar with its zero-stress (un-stretched) length L_0_ and stretched length L. The 1D strain and stress (for simplification) are defined by:

$$Strain=\frac{L-L_{0}}{L_{0}}$$ (1)

Stress:σ=YM∙ϵ (2)

where YM (Young’s modulus) is a commonly-used material stiffness parameter. Knowing zero-stress length of the bar is essential for its stress/strain calculations. Fig 2(B) and 2(C) gave bar illustrations of myocardium fiber (sarcomere) zero-stress diastole and systole length and its length when stressed in vivo. Sarcomere shortening is reflected by the shorter length (L_0-sys_) of the bar in Fig 2(C). This bar plot also shows how sarcomere shortening leads to increased ventricle stress and strain values (note the bar length L in (b) and (c) remains the same). Ventricle material models and 3D stress/strain calculations are given in 2.3 and 2.4.

Since ventricle is under pressure, its accurate zero-stress geometry is unknown under in-vivo conditions. Our model construction needs to start from zero-stress geometries (the reference geometries) with zero pressure and zero stress and strain assigned. It should be noted that zero-load and zero-stress are two different concepts, since the residual stress exists in myocardium tissues [16]. However, it is near impossible to obtain ventricle zero-stress geometry from in vivo CMR data. Thus, zero-load geometries were used in our model construction process as approximations to zero-stress geometries. Due to myocardium active contraction and relaxation, zero-stress sarcomere lengths are different in systole (shortened) and diastole (relaxed) phase. Two different zero-load (diastole and systole) geometries are needed to simulate ventricle diastole and systole phases, respectively. These models are called 2G models since two zero-load geometries are used [13]. The 2G model is our way to change ventricle zero-load geometry between systole and diastole phases. A 2G model is practically using two models (each with a different zero-load geometry) to complete the cardiac cycle.

In our 2G model construction process, a pre-shrink process was applied to in vivo ventricle CMR data with minimum volume to obtain the approximate two zero-load geometries so that when in vivo pressure was applied, the ventricle would regain its in vivo geometry [13]. Shrinking is achieved by shrinking each slice (short-axis direction) with an initial short-axis shrinking rate and by reducing the slice distances (long-axis direction) with an initial long-axis shrinking rate. However, if the slice was shrunk uniformly, the ventricle wall volume (the muscle) would become smaller, which should not happen. So the inner contour (inner wall of the ventricle) was shrunk more, the outer contour (ventricle outer wall) was shrunk a little less so that the ventricle wall volume was conserved. To get the zero-load diastole geometry, we started with a 2% shrinkage, construct the model, and apply the minimum pressure (begin-diastole) to see if the pressurized RV volume matches the CMR data. If not, we adjust the shrinkage, re-made the model, pressurize it and check again. The process is repeated until RV volume matches CMR volume with error < 0.5%. For the zero-load systole geometry, assuming a 10–15% sarcomere shortening, we started with a 15% shrinkage. The same process was repeated until the pressurized RV volume under end-systole pressure (higher than the minimum pressure) matched the CMR volume data. LV geometries were handled in similar way, with proper shrinkages determined corresponding to LV pressure conditions. The zero-load systole geometry is smaller reflecting shortened zero-stress sarcomere length. Fig 1 provided the zero-load diastole and systole geometries from one TOF patient.

In the 1G model construction process, only one zero-load geometry was used to stimulate the cardiac cycle, with maximum pressure corresponding to maximum ventricle volume and minimum pressure corresponding to minimum volume, respectively. In 1G models, end-filling state and begin-ejection state were the same with identical pressure, volume, and ventricle stress and strain conditions; the same were true for begin-filling and end-ejection. That means 1G models used wrong pressure conditions for end-filling and end-ejection. Results for end-filling and end-ejection from 1G models could not be right. Those were 1G model limitations.

### 2.3 Governing equations and material models

Ventricle stress and strain were solved from the governing equations given below:

$$\rho v_{i,tt}=\sigma_{ij,j},i,j=1,2,3;\;sum\;over\;j,$$ (3)

$$\varepsilon_{ij}=\frac{1}{2}(v_{i,j}+v_{j,i}+v_{\alpha ,i}v_{\alpha ,j}),\;i,j,\alpha =1,2,3,$$ (4)

$${p|}_{RV}=p_{RV}(t),{p|}_{LV}=p_{LV}(t)$$ (5)

where *σ* is the stress tensor (which has 6 components), *ε* is the strain tensor (6 components), *v* is displacement vector (3 components), *p* is ventricle pressure, and *ρ* is material density. The normal stress was assumed to be zero on the outer RV/LV surface and equal to the pressure conditions imposed on the inner RV/LV surfaces. Eqs (3) and (4) contain 15 unknown functions (stress: 6; strain: 6; displacement: 3), but only 9 equations (Eq (3) has 3 scalar equations; Eq (4) has 6 scalar equations). The other 6 equations come from material properties linking stress and strain together. To that end, the RV and LV materials were assumed to be hyperelastic, anisotropic, nearly-incompressible and homogeneous. The patch and scar materials were assumed to be hyperelastic, isotropic, nearly-incompressible and homogeneous. The nonlinear Mooney-Rivlin model was used to describe the nonlinear anisotropic and isotropic material properties. The strain energy function for the isotropic modified Mooney-Rivlin model is given by Tang et al. [4, 5, 13–15, 17–19]:

$$W=c_{1}(I_{1}-3)+c_{2}(I_{2}-3)+D_{1}[\exp\left(D_{2}(I_{1}-3)\right)-1]$$ (6)

where I_1_ and I_2_ are the first and second strain invariants given by,

$$I_{1}=\sum C_{ii},I_{2}=\frac{1}{2}(I_{1}^{2}-C_{ij}C_{ij})$$ (7)

*C =* [*C*_*ij*_] *= X*^*T*^*X* is the right Cauchy-Green deformation tensor, *X* = [*X*_*ij*_] = [a*x*_*i*_*/* a*a*_j_], (*x*_*i*_) is the current position, (*a*_*i*_) is the original position, *c*_*i*_ and *D*_*i*_ are material parameters chosen to match experimental or patient-specific CMR measurements. The strain energy function for the anisotropic modified Mooney-Rivlin model was obtained by adding an additional anisotropic term in Eq (6) (Tang et al. [4, 5, 13–15, 17–19]):

$$W=c_{1}(I_{1}-3)+c_{2}(I_{2}-3)+D_{1}[\exp\left(D_{2}(I_{1}-3)\right)-1]+\frac{K_{1}}{K_{2}}[exp{\left(I_{4}-1\right)}^{2}-1]$$ (8)

where *I*_4_ = *C*_*ij*_(*n*_*f*_)_*i*_(*n*_*f*_)_*j*_, *C*_*ij*_ is the Cauchy-Green deformation tensor, *n*_*f*_ is the fiber direction, *K*_1_ and *K*_2_ are material constants. With the strain energy function determined, the stress-strain relationship is given by:

$$\sigma_{ij}=\frac{\partial W}{{\partial \epsilon }_{ij}},i,j=1,2,3.$$ (9)

Since stress tensor is symmetric, Eq (9) only has 6 scalar equations. Now Eqs (3), (4) and (9) include 15 scalar equations to determine stress, strain and displacement, altogether 15 scalar functions.

**Fig 3:**
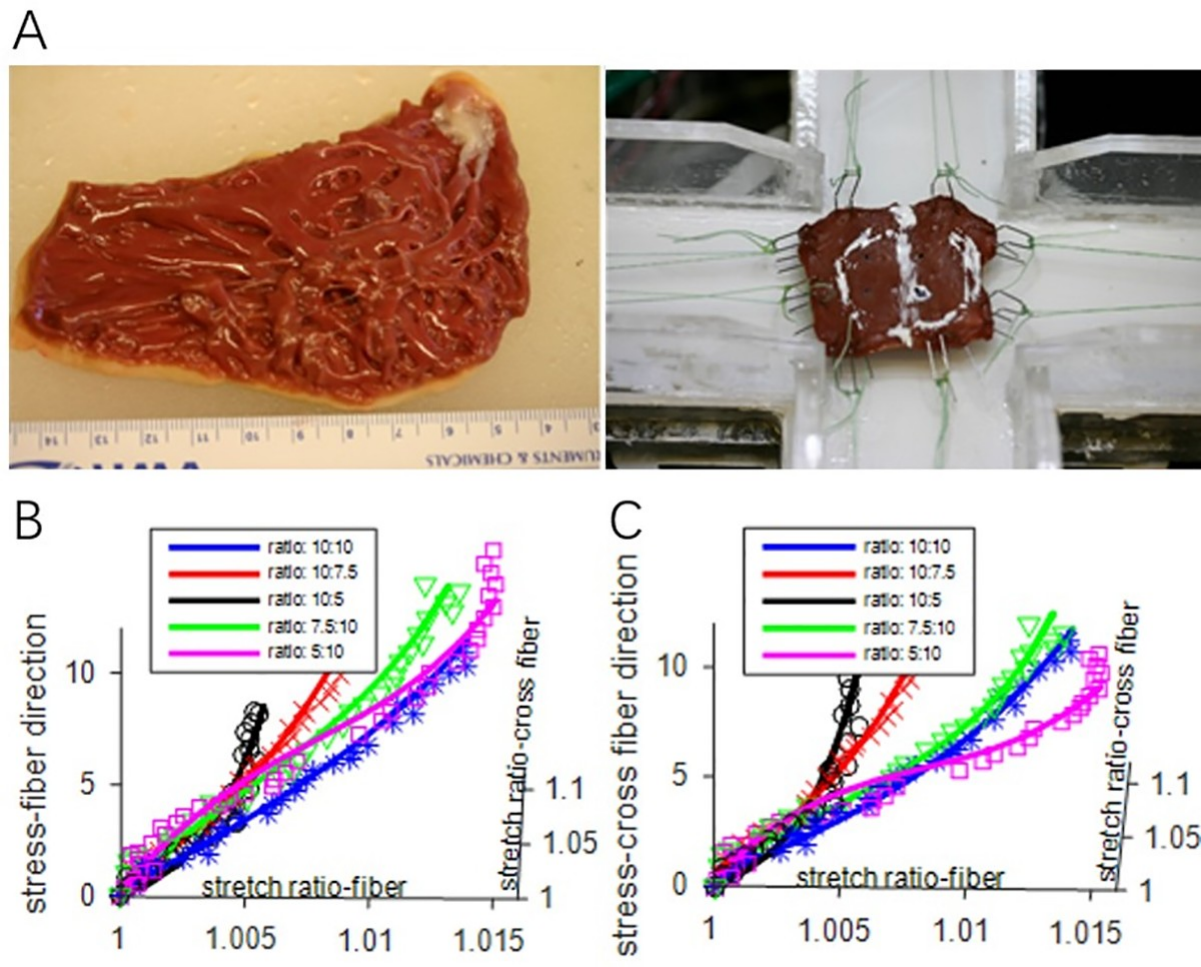
Biaxial mechanical testing and curve fitting with Mooney-Rivlin model. (A) A human right ventricle tissue sample mounted for biaxial test stress-stretch data recorded from 5 tests with fiber vs. cross-fiber stress ratio of 10:10, 7.5:10, 5:10, 10:7.5, and 10:5, respectively. (B) Stress-Strain Curve in fiber direction. (C) Stress-Strain Curve in cross-fiber direction.

Because tissue mechanical properties are essential for computational ventricular modeling, we generated the first complete biaxial mechanical data set for ventricular tissues using a cadaveric normal human heart sample obtained from NDRI—National Disease Research Interchange following required procedures and protocol approved by NDRI (see Fig 2) [14]. Detailed description of the custom biaxial testing device and method has been previously described [20, 21]. Myocardium tissue came from a 30-year-old female who was deceased because of head trauma. Approximately 2 cm by 2 cm full-thickness sections of the right free ventricular wall and thin left ventricular wall specimens were dissected from the cadaver heart obtained within 24 hours of harvest from the donor from National Disease Research Interchange (NDRI, Philadelphia, PA). Upon arrival the heart was perfused and stored in chilled cardioplegic solution to eliminate contraction of the muscle. For the right ventricular free wall, one epicardial sample was obtained due to the thinness of the wall. The preferred fiber direction was determined by placing four fiduciary (graphite chip) markers in the center of each sample, mounting it on the biaxial test machine via 16 stainless steel hooks with the base-apex direction aligned with the machine axes, then loading it equibiaxially and recording the displacement of the markers. The stiffest material axis will be determined by the deformation gradient, its orientation relative to the base-apex direction recorded, and the sample was trimmed to align with the material axes (~15mmx15mm), and the sample re-mounted on the biaxial test device. Five tests with different fiber-direction vs. cross-fiber-direction stress ratios were run and data were recorded for model fitting. We were able to choose parameter values in our modified Mooney-Rivlin model to fit our direct measurement of biaxial stress-strain data (see Fig 3).

It should be noted that the ex vivo biaxial testing data was only used to support the choice of our material model, i.e., the modified anisotropic Mooney-Rivlin model is able to represent ventricle tissue anisotropic material properties. The ex vivo biaxial testing data was not directly used in this paper. Patient-specific ventricle parameter values in the material model for each patient were determined using CMR-measured RV volume data and an iterative procedure [4, 5, 13–15, 17–19]. The parameter determination procedure and parameter values for all participants are given in the appendix.

The orientations of myofibrils cause the myocardial tissue to exhibit mechanical anisotropy [22]. Since patient-specific fiber orientation data was not available, we chose to construct a 2-layer RV/LV model and set fiber orientation angles using the fiber angles published by Hunter et al. (Fig 1) and available human data [7,23]. In our 2-layer models, left ventricle (LV) fiber orientation was approximately -60^o^ (relative to circumferential direction) at the epicardium and +80^o^ at the endocardium. RV fiber orientation was set at -45^o^ at the epicardium and +40^o^ at the endocardium [7, 23]. Fiber orientations could be adjusted when patient-specific data becomes available.

### 2.4 Solution methods

**Fig 4:**
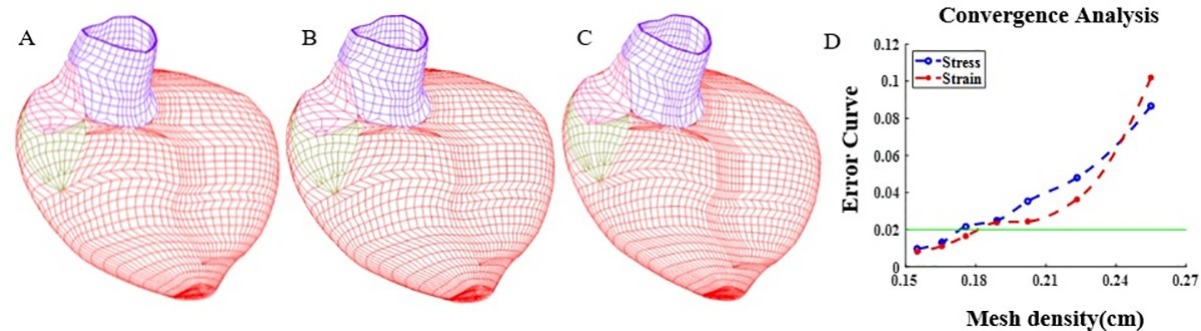
Mesh refinements and error plots using one sample. A. Mesh with 20630 elements (Mesh density: 0.189cm). B. Mesh with 25550 elements (Mesh density: 0.176cm). C. Mesh with 30554 elements (Mesh density: 0.166cm). D. Stress and strain relative L1 norm error plots.

The RV/LV computational models were constructed for the 18 participants and solved by ADINA (ADINA R&D, Watertown, MA, USA). Model construction and solution procedures were reported previously [13–15]. Parameters in the Mooney-Rivlin models were adjusted iteratively until good agreement between the computational and CMR-measured volume data was found (error<0.2%). Mesh analysis was performed by reducing mesh density by 10–15% incrementally in each dimension until solutions became mesh independent, i.e., differences of solutions (stress/strain) between two consecutive meshes became smaller than the set tolerance (2%). Mesh density is the step size for an edge (line) of a volume ADINA uses to generate mesh for the given volume. Since each RV/LV model consisted of hundreds of manually-constructed small volumes and mesh was generated by ADINA with specified mesh density (or number of divisions for each edge of the volume), mesh density was refined by increasing number of divisions in each edge for a given small volume. That was done for all volumes of the given RV/LV model. Fig 4 gave three consecutive meshes refinement example and stress/strain error plots showing convergence. L1 norm was to calculate stress and strain errors between two consecutive meshes. The average of mesh density for all volumes for a given mesh was used to make Fig 4D. When mesh was settled for a model, simulation procedures were continued for 3 periods when differences in the solutions between the last two periods became less than 0.1%. The solutions for the last period were accepted for analysis. Three periods were used since a) our simulation started from zero pressure with zero stress/strain so Period 2 solutions differed from Period 1 solutions considerably; b) the solutions became periodic from the second period so Period 2 and Period 3 solutions were almost identical.

### 2.5 Data extraction and preparation for comparative and statistical analysis

Since stress and strain are tensors, maximum principal stress and strain (both are scaler functions) were used to as the representative stress/strain variables for comparative analyses. For each ventricle model, 100 evenly-spaced points were assigned for each slice. Maximum principal stress and strain values on those points from all the slices were extracted from the model and their mean values were recorded for analysis [14]. Average right ventricle stress and strain values at begin-filling (BF), end-filling (EF), begin-ejection (BE) and end-ejection (EE) were recorded for analyses. The Wilcoxon rank sum test for unpaired observations was used to compare stress and strain differences between different groups. The Wilcoxon signed rank test for paired treatments was selected to compare stress and strain values at the 4 selected key time points (BF, EF, BE, EE). Spearman rank correlation was used to calculate correlation between computational stress/strain and CMR-measured volume data.

For a given patient, it is of clinical importance to predict if the patient would have better post-PVR outcome based on pre-surgery data. For our study, this comes to determine if a patient belongs to BG (better outcome group) or WG (worse-outcome group) based on patient’s baseline data, without knowing post-PVR outcome. To predict if a patient belongs to BG or WG using pre-PVR data, we used the logistic regression model:

$$logit(Pr(y_{i}=1))=\beta_{0}+\beta_{1}W_{i}$$ (10)

where y_i_ denotes the ith patient and W_i_ denotes predictor W’s value from that patient. y_i_ = 1 (positive outcome) indicates that the model predicted that the patient belongs to BG, while y_i_ = 0 (negative outcome) indicates that the model predicted that the patient belongs to WG. RV stress, strain, RVEDVi, EVESVi, gender, EF, and age was used as the predictors to check their prediction accuracy. For “sex” variable, male was represented with 1 and female was represented with 0 in the analysis. To stabilize the result, a 5-fold cross-validation procedure was adopted and repeated 200 times automatically with a random partition of training and testing groups each. The gradient descent method was used to obtain the coefficients by the training data, and the logistic regression model obtained from the training set was applied to the testing set to make predictions. Prediction results were compared to actual post-PVR outcome to calculate prediction accuracy, sensitivity, and specificity defined as:

$$Accuracy=\frac{TP+TN}{TP+FP+TN+FN}$$ (11)

$$Sensitivity=\frac{TP+TN}{TP+FP+TN+FN}$$ (12)

$$Specificity=\frac{TP+TN}{TP+FP+TN+FN}$$ (13)

where TP is the number of true positive outcomes, TN is the number of true negative outcomes, FP is the number of false positive outcomes, and FN is the number of false negative outcomes. Receiver operating characteristic (ROC) curve together with false positive rate and true positive rate were determined to qualify classification efficiency.

## Results

### 3.1 2G model stress/strain results showed considerable improvements over 1G results

Being able to provide end-diastole and end-systole stress/strain values is one of the major improvements of 2G models over 1G models since 1G models did not have those results. Results from 2G models included stress/strain values at begin-filling (BF, corresponding to minimum volume with minimum pressure), end-filling (EF, corresponding to maximum volume with end-diastole pressure), begin-ejection (BE, corresponding to maximum volume with maximum pressure) and end-ejection (EE, corresponding to minimum volume with end-systole pressure). Results from 1G models included stress/strain values at begin-filling (BF, corresponding to minimum volume with minimum pressure) and begin-ejection (BE, corresponding to maximum volume with maximum pressure). 1G models treated EF and BE (and EE and BF as well) as identical since 1G models did not use end-diastole and end-systole pressure conditions at end-diastole and end-systole, respectively.

Tables 2 and 3 summarized the stress and strain values from the 18 participants at BF, EF, BE, and EE, respectively. At end-ejection, 2G mean stress value was 321.4% higher than that of 1G models (14.54±7.41vs. 3.45±1.62, p = 0.0002). 2G mean strain values was 230% higher than that of 1G models (0.099±0.015 vs. 0.030±0.009, p = 0.0002). At begin-ejection, mean stress of 2G models was 4.9% higher than that of 1G models (61.49±26.83kPa vs. 58.60±25.41, p = 0.0010). Mean strain of 2G models was 23.1% than that of 1G models (0.548±0.126 vs. 0.445±0.125, p = 0.0002). The differences at end-ejection was much greater because when pressure and deformation were smaller, the stress/strain differences caused by the zero-load geometries became far more noticeable. Differences in different groups can be observed from Tables 2 & 3.

**Table 2:** Average RV stress (Stress-P_1_) from 6 healthy volunteers and 12 TOF patients.

Groups		Patients	1G models		2G models			
			BF(kPa)	BE(kPa)	BF(kPa)	EF(kPa)	BE(kPa)	EE(kPa)
Healthy Group		P1	1.91	48.18	1.91	22.38	49.57	8.07
		P2	1.90	42.13	1.90	17.40	45.13	7.89
		P3	1.68	33.37	1.68	14.79	37.55	7.60
		P4	2.35	39.52	2.35	17.15	39.89	8.48
		P5	2.76	40.42	2.76	17.33	45.19	9.13
		P6	2.14	47.15	2.14	19.99	50.21	10.06
		HG Mean	2.12	41.80	2.12	18.17	44.59	8.54
		±SD	±0.39	±5.43	±0.39	±2.64	±5.07	±0.92
TOF Group	Better Outcome Group	P7	2.84	41.77	2.84	19.17	43.29	13.87
		P8	4.15	54.51	4.15	25.36	55.70	17.68
		P9	4.90	87.25	4.90	35.16	91.36	18.18
		P10	2.94	57.38	2.94	22.80	62.87	13.13
		P11	1.51	27.68	1.51	11.15	26.02	5.98
		P12	2.10	36.08	2.10	14.90	38.34	7.74
		BG Mean	3.07	50.78	3.07	21.42	52.93	12.76
		±SD	±1.26	±21.06	±1.26	±8.48	±22.86	±5.02
	Worse Outcome Group	P13	5.78	116.33	5.78	48.87	122.54	22.04
		P14	3.85	49.81	3.85	21.11	52.00	14.42
		P15	5.77	76.22	5.77	29.93	80.48	30.30
		P16	4.14	98.91	4.14	37.01	108.67	17.12
		P17	6.81	92.57	6.81	39.84	91.76	29.37
		P18	4.67	65.52	4.67	29.25	66.21	20.63
		WG Mean	5.17	83.23	5.17	34.34	86.94	22.32
		±SD	±1.14	±24.11	±1.14	±9.68	±26.29	±6.42
	TOF Group Mean		4.12	67.00	4.12	27.88	69.94	17.54
	± SD		±1.59	±27.44	±1.59	±10.99	±29.45	±7.42
All HG+TG Mean±SD			3.45	58.60	3.45	24.64	61.49	14.54
			±1.62	±25.41	±1.62	±10.12	±26.83	±7.41

**Table 3:** Average RV strain (Strain-P_1_) from 6 healthy volunteers and 12 TOF patients.

Groups		Patients	1G models		2G models				
			BF(kPa)	BE(kPa)	BF(kPa)	EF(kPa)	BE(kPa)		EE(kPa)
Healthy Group		P1	0.031	0.637	0.031	0.648		0.728	0.100
		P2	0.026	0.531	0.026	0.531		0.636	0.082
		P3	0.032	0.523	0.032	0.530		0.612	0.108
		P4	0.016	0.486	0.016	0.483		0.582	0.089
		P5	0.042	0.544	0.042	0.544		0.611	0.084
		P6	0.038	0.575	0.038	0.564		0.669	0.120
		HG Mean	0.308	0.549	0.308	0.550		0.640	0.097
		±SD	±0.009	±0.052	±0.009	±0.054		±0.052	±0.015
TOF Group	Better Outcome Group	P7	0.021	0.339	0.021	0.338		0.442	0.093
		P8	0.023	0.278	0.023	0.280		0.376	0.092
		P9	0.032	0.462	0.032	0.476		0.591	0.111
		P10	0.042	0.491	0.042	0.489		0.601	0.122
		P11	0.027	0.414	0.027	0.421		0.528	0.093
		P12	0.033	0.368	0.033	0.370		0.472	0.093
		BG Mean	0.029	0.392	0.029	0.396		0.502	0.101
		±SD	±0.008	±0.079	±0.008	±0.082		±0.088	±0.012
	Worse Outcome Group	P13	0.051	0.609	0.051	0.600		0.724	0.124
		P14	0.028	0.335	0.028	0.328		0.424	0.078
		P15	0.018	0.217	0.018	0.220		0.322	0.084
		P16	0.037	0.567	0.037	0.562		0.698	0.120
		P17	0.028	0.331	0.028	0.333		0.428	0.107
		P18	0.022	0.310	0.022	0.278		0.411	0.087
		WG Mean	0.031	0.395	0.031	0.387		0.501	0.100
		±SD	±0.012	±0.156	±0.012	±0.156		±0.167	±0.020
	TG Mean		0.030	0.393	0.030	0.391		0.501	0.100
	±SD		±0.010	±0.118	±0.010	±0.119		±0.127	±0.016
All HG+TG Mean±SD			0.030	0.445	0.030	0.444		0.548	0.099
			±0.009	±0.125	±0.009	±0.126		±0.126	±0.015

### 3.2 Mean RV stress value at begin-ejection is higher than that at end-filling

1G models treated end-filling and begin-ejection as identical (they were the same time point connection systole and diastole phases) so 1G models could not provide the stress/strain differences between end-filling and begin-ejection. In real cardiac cycle, active contraction happens between end-filling and begin-ejection (it is called the isovolumic phase), sarcomere zero-stress length shortens, ventricle stress/strain and pressure increase while its volume is kept unchanged (both valves are closed). 2G models actually have stress/strain values at end-filling and begin-ejection from their diastole and systole phases, respectively. Mean stress of all 18 cases at begin-ejection was 150% higher than end-filling mean stress value (61.49±26.83kPa vs. 24.64±10.12kPa, p = 0.0002). Begin-ejection mean strain value was 23.26% higher than that of end-filling (0.548±0.126 vs. 0.444±0.126, p = 0.0002). The higher stress and strain at begin-systole is caused by sarcomere shortening in iso-volume contraction (active contraction). The average wall stress and strain values reach their maximum values in begin-ejection due to the high pressure and smaller zero-load configurations reflecting sarcomere shortening.

### 3.3 Mean RV stress value in begin-filling is lower than that of end-ejection

Similar to isovolumic contraction, isovolume relaxation happens between end-ejection and begin-filling from, 2G models are able to calculate RV stress and strain at end-ejection and begin-filling with their respective systolic and diastolic zero-load geometries and corresponding pressure conditions. Mean stress value at end-ejection was 321.45% higher than that of begin-filling (14.54±7.41kPa vs. 3.45±1.62kPa, p = 0.0002). Mean strain value in begin-filling was 233.3% higher than that of begin-filling mean strain value (0.030±0.009 vs. 0.099±0.015, p = 0.0002). The average wall stress and strain values reach their minimum values at begin-filling due to the lower pressure and sarcomere relaxation.

### 3.4 TOF group had higher stress than healthy group and healthy group had higher strain than TOF group

**Fig 5:**
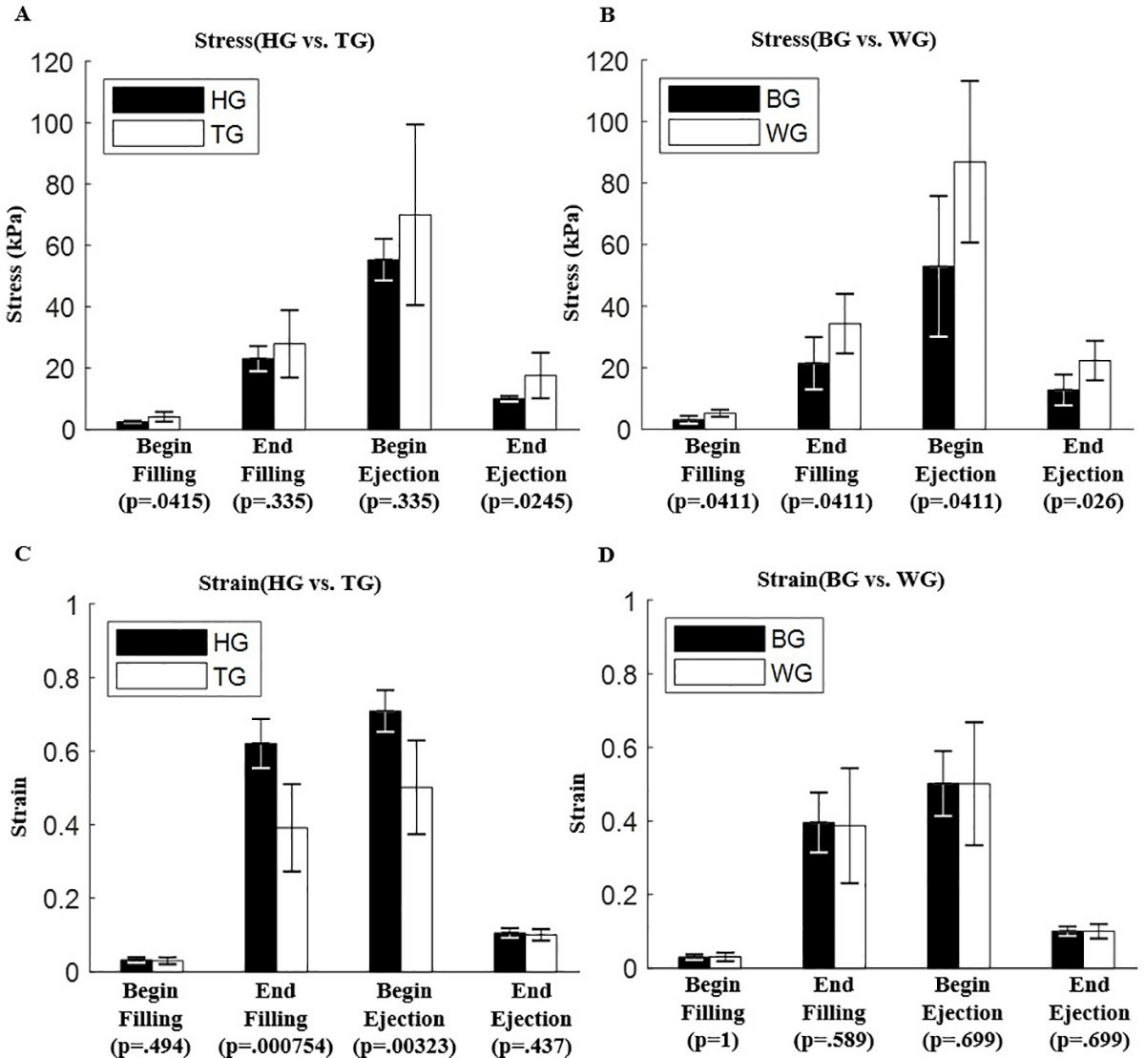
Stress/Strain group comparisons at begin-filling, end-filling, begin-ejection and end-ejection. (A) Comparison between HG and TG stress. (B) Comparison between BG and WG stress. (C) Comparison between HG and TG strain. (D) Comparison between BG and WG strain.

Table 4 and Fig 5 provided stress/strain comparison between different groups at BF, EF, BE and EE with their respective p-values. In general, mean stresses from TOG group were higher than that from the healthy group. Statistically significant stress differences between HG and TG were found at begin-filling and end-ejection. At begin-filling, TG mean stress value was 94.34% higher than that of HG (4.12±1.59kPa vs. 2.12±0.39kPa, p = 0.0414). At end-ejection, TG mean stress value was 105.3% higher than that of HG (17.54±7.42kPa vs. 8.54±0.92kPa, p = 0.0245). Mean strain values of healthy individuals is 40.7% higher than that of TOF patients at end-filling (0.550±0.055 vs 0.391±0.119, p = 0.0008) and 28% higher than that of TOF patients at begin-ejection ((0.640±0.052 vs 0.501±0.127, p = 0.0032). Strain differences are not found in end-filling and begin-ejection. Higher strain level from HG indicated that healthy ventricles had better contractibility and TG ventricles contracted less.

**Table 4:** Stress/Strain group comparisons between healthy and TOF group and between BG and WG at BF, EF, BE and EE.

Group	BF(kPa)	EF(kPa)	BE(kPa)	EE(kPa)
Comparison of mean stress between HG and TG				
HG	2.12±0.39	18.17±2.64	44.59±5.07	8.54±0.92
TG	4.12±1.59	27.88±10.99	69.94±29.45	17.54±7.42
p-value	0.0414	0.3355	0.3355	0.0245
Comparison of mean strain between HG and TG				
HG	0.031±0.009	0.550±0.055	0.640±0.052	0.097±0.015
TG	0.030±0.009	0.391±0.119	0.501±0.127	0.100±0.016
p-value	0.4936	0.0008	0.0032	0.4371
Comparison of mean stress between BG and WG				
BG	3.07±1.26	21.42±8.48	52.93±22.86	12.76±5.02
WG	5.17±1.14	34.34±9.68	86.94±26.29	22.32±6.42
p-value	0.0411	0.0411	0.0411	0.0260
Comparison of mean strain between BG and WG				
BG	0.030±0.008	0.396±0.081	0.502±0.088	0.101±0.013
WG	0.031±0.011	0.387±0.156	0.501±0.167	0.100±0.020
p-value	1.0000	0.5887	0.6991	0.6991

### 3.5 RV stress of WG was much higher than that of BG

At begin-ejection, mean RV stress of WG is 57.4% higher than that of BG (86.94±26.29kPa vs. 52.93±22.86 kPa; p = 0.041). Differences at other three time points were similar. At end-ejection, mean stress of WG is 74.9% higher than that of BG (22.32±6.42kPa vs. 12.76±5.02kPa; p = 0.026). At begin-filling, mean stress of WG is 68.4% higher than that of BG (5.17±1.14kPa vs. 3.07±1.26kPa; p = 0.041). At end-filling, mean stress of WG is 60.3% higher than that of BG (34.34±9.68kPa vs. 21.42±8.48kPa; p = 0.041). Strain differences between the two groups were not statistically significant.

### 3.6. RV stress is the best predictor for post-PVR outcome among the 13 selected parameters

Table 5 listed the AUC, prediction accuracy, sensitivity and specificity of the 13 selected predictors (7 if not counting different time stress/strain as different predictors), with stress and strain at BF, EF, BE and EE all included for comparisons. RV stress had the best AUC (0.8135) and prediction accuracy (0.8208). Among the begin-filling, end-filling begin-ejection and end-ejection time points, end-filling stress had the best prediction accuracy at 0.8208, noticeably better than its accuracies at BE (0.7638), BF (0.8054), and EE (0.7879). Other the other hand, end-ejection stress had best AUC value at 0.8135. Prediction accuracy of age was 0.6542, just below the stress predictors. RVEDVi and RVESVi had prediction accuracies at 0.6296 and 0.5675, respectively. Prediction accuracy of ejection fraction was only 0.5000. Ejection fraction, sex and strain all had accuracies at about 0.5, indicating poor predicting power.

**Table 5:** RV stress is the best predictor for post-PVR outcome among the 7 selected parameters.

Predictor	BG	WG	Sensitivity	Specificity	AUC	Accuracy	
Stress_End_Filling	21.42±8.48	34.34±9.68	0.8217	0.8200	0.7437		0.8208
Stress_Begin_Filling	3.07±1.26	5.17±1.14	0.6658	0.9450	0.7876		0.8054
Stress_End_Ejection	12.76±5.02	22.32±6.42	0.9258	0.6500	0.8135		0.7879
Stress_Begin_Ejection	52.93±22.86	86.94±26.29	0.7283	0.7992	0.7245		0.7638
Age	31.1±17.2	42.7±16.2	0.5467	0.7617	0.5701		0.6542
RVEDVi	197.69±20.09	214.57±54.91	0.8133	0.4458	0.4589		0.6296
RVESVi	112.69±18.87	128.98±46.36	0.9708	0.1642	0.4063		0.5675
Strain_Begin_Ejection	0.502±0.088	0.501±0.167	0.9967	0.0050	0.1924		0.5008
EF	42.3±5.4	40.8±8.4	1.0000	0.0000	0.3364		0.5000
Sex	-	-	1.0000	0.0000	0.1913		0.5000
Strain_Begin_Filling	0.030±0.008	0.031±0.011	0.9975	0.0025	0.2189		0.5000
Strain_End_Filling	0.396±0.081	0.387±0.156	0.0017	0.9983	0.2311		0.5000
Strain_End_Ejection	0.101±0.013	0.100±0.020	1.0000	0.0000	0.1858		0.5000

### 3.7 Correlations between RV stress/strain and MRI-derived parameters

Correlation analyses were performed to determine whether RV stress/strain were associated with MRI-derived parameters including ejection fraction change (△EF), RV end-diastole volume and end-diastole volume index. Results are given in Table 6. Using the 2G model results, RV EF change correlated negatively with end-filling stress (r = -0.650, p = 0.026) and begin-ejection stress(r = -0.608, p = 0.040). Other correlations were not found. It is worth noting that while end-filling and begin-ejection stress/strain mean values differ considerably, their correlation results were similar.

**Table 6:** Stress/Strain correlations with RVEF change, RV EDV and RV EDVi.

	Mean±SD	[r, p]	△EF	RV EDV	RV EDVi
Stress_end_filling	27.88± 10.99kPa	r	-0.650	0.469	-0.056
		p	0.026	0.128	0.869
Stress_begin_ejection	69.94± 29.45kPa	r	-0.608	0.454	-0.119
		p	0.040	0.140	0.762
Strain_end_filling	0.391 ±0.119	r	0.3287	-0.3497	-0.4895
		p	0.2974	0.2660	0.1096
Strain_begin_ejection	0.501 ±0.127	r	0.3007	-0.3287	-0.4615
		p	0.3425	0.2974	0.1338

## Discussions

### 4.1. 2G models can provide more accurate stress/strain calculations with proper zero-load ventricle geometries, especially end-diastole and end-systole stress/strain conditions

It is self-evident from the results presented that 2G models could give end-diastole and end-systole stress/strain conditions that 1G models could not produce due to model limitations. Using correct reference frame is a basic requirement for correct stress/strain calculations [13–15]. 2G model could be considered an improvement over 1G model based on stress/strain theoretical definitions in biomechanics. The ultimate gold standard to prove that 2G models are more accurate than 1G models requires the knowledge of zero-stress reference ventricle diastole and systole geometries which is extremely difficult to have if after all possible.

One limitation of 1G models is that not only 1G models do not change zero-load geometry between systole and diastole phases, they also use wrong pressure conditions at end-filling and end-ejection. As a result, end-filling and end-ejection stress/strain calculations using 1G models would have large errors and could be fairly misleading.

Various circumstances and applications may require accurate ventricle stress/strain calculations, such as measuring how hard the ventricle is working, energy consumed, and designing ex vivo experiments studying myocardium material properties. 2G models could provide more realistic stress/strain calculations in those cases.

Table 7 gave post-PVR outcome prediction results using 1G models. It should be noted that 1G models had only two extreme time points in a cardiac cycle (begin-filling and begin-ejection) since (end-filling, begin-ejection) and (end-ejection, begin-filling) were modeled as identical points. Our 1G and 2G begin-filling conditions were the same. So Table 7 had prediction results for only one time point. 1G prediction results were in line with 2G results given in Table 5. In particular, prediction accuracy (0.8141) using 1G Begin-Ejection stress was better than that (0.7638) using 2G Begin-Ejection stress, and slightly lower than the best 2G accuracy (0.8206) using 2G End-Filling stress. It should be noted that whether stress from 2G model was a better predictor or not does not prove or disprove if 2G models gave better stress calculators. Those two were separate issues.

**Table 7:** Post-PVR outcome predictions using 1G models.

Predictor	BG	WG	Sensitivity	Specificity	AUC	Accuracy
Stress_Begin_Ejection	50.78±21.06	83.23±24.11	0.8098	0.8183	0.7370	0.8141
Strain_Begin_Ejection	0.392±0.079	0.395±0.156	0.0000	1.0000	0.1991	0.5000

### 4.2. Our mechanical stress/strain study compared to other TOF patient studies in the literature

Our previous publications indicated that mechanical stress could be used to differentiate TOF patients with better post-PVR outcome from TOF patients with worse post-PVR outcome [4, 5]. While our purpose of this modest paper was mainly to provide some benchmark stress/strain data for TOF patients and healthy and their comparisons, many investigators have made interesting studies, often based on morphological features with aims for clinical applications. Leonardi et al. used an unbiased shape analysis framework and linear regression to analyze 38 rToF patients [24]. They found that regurgitation severity was significantly associated with RV dilatation (p = 0.01) and associated with bulging of the outflow tract (p = 0.07) and a dilatation of the apex (p = 0.08). Toussaint et al. pushed out an integrated platform which aims at helping researchers and clinicians to visualize and process dynamic image data, as well as evaluate simulation results. They used personalized simulation of the Tetralogy of Fallot to illustrate their platform [25]. Geva et al. performed a study using clinical and laboratory data of 100 consecutive patients with repaired TOF (median age 21) to identify independent factors associated with impaired clinical status in late survivors of tetralogy of Fallot (TOF) repair [26]. Among RV variables, a lower RV EF was the strongest independent factors associated with poor clinical status (odds ratio (OR) = 2.41 for 10% decrease, p = 0.01). A study by Lee et al. also revealed the association between severity pulmonary regurgitation and right ventricle volume index [27]. Some other studies also states the correlation between ventricle shape and pulmonary regurgitation indexed with body surface area [28]. Compared to healthy ventricles, the motion and shape features obtained from TOF ventricles also showed high variability and abnormality, indicating the potential use as TOF progression indicators such as RV dilation [29]. Interestingly, similar phenomenon was also found within our results: compared to healthy volunteers, TOF patients RV stress showed higher variability.

In our analysis, the morphology features such as RV EDVi and ESVi of TOF patients showed weak capability in post-PVR outcome prediction, which, however, didn’t deny the whole usage of ventricle morphology features. In our TOF population, RV EDVI and ESVI in BG and WG had no statistical significant differences (p = 0.937, p = 0.818). More importantly, compared to TOF population discussed in the references [24–29], 12 tested subjects in our analysis had much higher RV EDVi and ESVi and lower RV EF, indicating TOF patients in our analysis suffering from more severe RV failure and dilation. The severity in pathology should be considered.

### 4.3. Potential clinical applications

It has been a challenge for cardiovascular researchers to know before-hand which patients would have better post-PVR outcome. Our finding that ventricle stress had the best prediction accuracy (0.8208) in differentiating patients with better post-PVR from patients with worse post-PVR outcome leads to the potential in using mechanical analysis in patient screening and treatment strategies. Knowing that better-outcome patients had lower RV stress level, novel surgical plans could be designed in reducing RV stress level for possible better outcome.

Comparison between healthy and TOF patients revealed that TOF patients have in general higher stress and lower strain levels. That suggested in surgical treatment considerations, the surgeon could introduce novel strategies to reduce ventricle stress and increase ventricle strain (linked to ventricle contractivity) levels. Dr. del Nido proposed a novel PVR surgical strategy to use smaller patch with aggressive scar trimming to improve post-PVR outcome [30,31]. In our clinical trial (NIH 5P50HL074734, Geva and del Nido), 64 patients with repaired tetralogy of Fallot, and who fulfilled defined criteria for PVI were randomly assigned to undergo either PVI alone (n = 34) or PVI with surgical RV remodeling (n = 30). However, no significant difference was observed in the primary outcome (change in RV ejection fraction, -2±7% in PVI alone (Fig 1A), vs. -1±7% in PVI with RV remodeling; P = 0.38) or in any secondary outcomes [31]. Our modeling study indicated that PVR with a smaller patch and more aggressive scar removal led to reduced stress conditions and may lead to improved recovery of RV functions (2–3% EF improvement) [15, 32]. However, results from modeling study were with simplified and idealized conditions and need to be validated by large-scale patient studies.

### 4.4. Model limitations and future directions

Limitations in our work include the following: a). Iso-volumic contraction and relaxation were skipped since they involve continuous reference frame change caused by sarcomere zero-stress length change that our current method is not able to handle; b). valve mechanics were not included; c) Patient-specific fiber orientation were unavailable to us. Patient-specific TOF RV/LV fiber orientations should be acquired and used in our future models; d). a pre-shrink method was applied to in vivo ventricle geometry obtained from CMR data to obtain approximate zero-load geometries and used in our models, which could alter the shape of ventricle when in-vivo pressure was applied. Ohayon et al. and Speelman et al. have developed good method to obtain zero-load geometries which will be considered in our future effort [33,34].

Direct measurements of ventricle in-vivo stress and zero-load sarcomere length are nearly impossible. Ventricle strain measured by some medical devices are often calculated based on some in vivo ventricle reference frames (with either maximum or minimum geometry). Those strains are different from the strain calculated in this paper based on zero-load geometries. Our model is an improvement over the old 1G models [13].

## Supporting information

S1 Appendix Patient-Specific myocardium material parameter value determination. (DOCX)

S1 Table Parameters in Mooney-Rivlin model for myocardium (c_2_ = 0 kPa, D_2_ = 3.0, K_2_ = 3.0). BE: Begin-Ejection; EE: End-Ejection; BF: Begin-Filling; EF: End-Filling.
(DOCX)

## Abbreviations

APG: All Patient Group
AUC: Area under the receiver operating characteristic curve
BG: Better-outcome patient group
CMR: Cardiac magnetic resonance
EF: Ejection fraction
EDV: End-diastole volume
ESV: End-systole volume
HG: Healthy group
LV: Left ventricle
Strain-P_1_: Maximum principal strain
Stress-P_1_: Maximum principal stress
PVR: Pulmonary Valve Replacement
RV: Right ventricle
RVOT: Right Ventricular Outflow Tract
RVEF: RV ejection fraction
ΔEF: RV EF change
RVEDVi: Right Ventricle End Diastolic Volume index
RVESVi: Right Ventricle End Systolic Volume index
ROC curve: Receiver operating characteristic curve
TOF: Tetralogy of Fallot
TG: Tetralogy of Fallot group
WG: Worse-outcome Patient Group

## References

[1] Waien SA, Liu PP, Ross BL, Williams WG, Webb GD, McLaughlin PR. Serial follow-up of adults with repaired tetralogy of Fallot. Journal of Am Coll Cardiol. 1992; 20: 295–300. 1634663 10.1016/0735-1097(92)90093-3

[2] Therrien J, Siu SC, McLaughlin PR. Pulmonary valve replacement in adults late after repair of tetralogy of Fallot: are we operating too late? J Am Coll Cardiol. 2000; 36:1670–5. 10.1016/s0735-1097(00)00930-x 11079675

[3] Vliegen HW, Van Straten A, De Roos A, Roest AA, Schoof PH, Zwinderman AH, et al Magnetic resonance imaging to assess the hemodynamic effects of pulmonary valve replacement in adults late after repair of tetralogy of Fallot, Circulation, 2002; 106:1703–1707. 10.1161/01.cir.0000030995.59403.f8 12270866

[4] Tang D, Yang C, Geva T, Del Nido PJ. Right ventricular local longitudinal curvature as a marker and predictor for pulmonary valve replacement surgery outcome: An initial study based on preoperative and postoperative cardiac magnetic resonance data from patients with repaired tetralogy of Fallot. The Journal of Thoracic and Cardiovascular Surgery, 2014;147: 537–538. 10.1016/j.jtcvs.2013.08.054 24100105 PMC3957093

[5] Tang D, Zuo H, Yang C, Wu Z, Huang X, Rathod RH, et al, Comparison of Right Ventricle Morphological and Mechanical Characteristics for Healthy and Patients with Tetralogy of Fallot: An In Vivo MRI-Based Modeling Study, Molecular & Cellular Biomechanics, MCB, 2017; 14: 137–151. 30147632 10.3970/mcb.2017.014.137 PMC6103627

[6] McCulloch AD, Waldman L, Rogers J, Guccione JM. Large-scale finite element analysis of the beating heart, Critical Rev. in Biomed Eng, 1992; 20(5,6): 427–449. 1486784

[7] Hunter PJ, Pullan AJ, Smaill BH. Modeling total heart function, Annu Rev Biomed Eng., 2003; 5:147–77. 10.1146/annurev.bioeng.5.040202.121537 14527312

[8] Holmes JW, Lumens J, Clinical Applications of Patient-Specific Models: The Case for a Simple Approach. J Cardiovasc Transl Res. 2018;11(2):71–79. 10.1007/s12265-018-9787-z Epub 2018 Feb 16. 29453747 PMC5910244

[9] Costa KD, Takayama Y, McCulloch AD, Covell JW. Laminar fiber architecture and three-dimensional systolic mechanics in canine ventricular myocardium, Am J Physiol. 1999; 276:595–607. 10.1152/ajpheart.1999.276.2.H595 9950861

[10] Gan Y, Chen Q, Zhang S, Ju S, Li ZY. MRI-based strain and strain rate analysis of left ventricle: a modified hierarchical transformation Model, BioMedical Engineering OnLine 2015, 14, S1:S9 10.1186/1475-925X-14-S1-S9 Epub 2015 Jan 9, 2015. 25602778 PMC4306125

[11] Saber NR, Gosman AD, Wood NB, Kilner PJ, Charrier CL, Firman DN. Computational flow modeling of the left ventricle based on in vivo MRI data: initial experience, Annals of Biomed. Engng., 2001; 29:275–283. 10.1114/1.1359452 11339325

[12] Axel L. Biomechanical dynamics of the heart with MRI.Annu. Rev. Biomed. Eng., 2000;4: 321–347 10.1146/annurev.bioeng.4.020702.153434 12117761

[13] Tang D, Del Nido PJ., Yang C, Zuo H, Huang X, Rathod RH, et al, Patient-Specific MRI-Based Right Ventricle Models Using Different Zero-Load Diastole and Systole Geometries for Better Cardiac Stress and Strain Calculations and Pulmonary Valve Replacement Surgical Outcome Predictions, PLoS One, 2016:e0162986 10.1371/journal.pone.0162986 27627806 PMC5023146

[14] Tang D, Yang C, del Nido PJ, Zuo H, Rathod RH, Huang X, et al, Mechanical stress is associated with right ventricular response to pulmonary valve replacement in patients with repaired tetralogy of Fallot mechanical stress, Journal of Thoracic and Cardiovascular Surgery, 2015;151:687–694. 10.1016/j.jtcvs.2015.09.106 26548998 PMC4761474

[15] Tang D, Yang C, Geva T, del Nido PJ. Patient-Specific MRI-Based 3D FSI RV/LV/Patch Models for Pulmonary Valve Replacement Surgery and Patch Optimization. ASME. J Biomech Eng. 2008;130(4):041010-041010-10. 10.1115/1.2913339 18601452 PMC2918812

[16] Holzapfel GA, Ogden RW. Constitutive modelling of passive myocardium: a structurally based framework for material characterization. Philosophical Transactions of the Royal Society of London A: Mathematical, Physical and Engineering Sciences, 2009, 367(1902): 3445–3475. 10.1098/rsta.2009.0091 19657007

[17] Fan R, Tang D, Yao J, Yang C, Xu D, 3D Echo-Based Patient-Specific Computational Left Ventricle Models to Quantify Material Properties and Stress/Strain Differences Between Ventricles With and Without Infarct, Comput Model Eng Sci. 2014; 99: 491–508. 25663830 PMC4319570

[18] Fan L, Yao J, Yang C, Wu Z, Xu D, Tang D. Material stiffness parameters as potential predictors of presence of left ventricle myocardial infarction: 3D echo-based computational modeling study, Biomed Eng Online. 2016 4 5;15:34 10.1186/s12938-016-0151-8 27044441 PMC4820947

[19] Fan L, Yao J, Yang C, Xu D, Tang D, Using 3D Echo-based Modeling to Quantify in Vivo Ventricle Material Properties: A Multi-patient Study, Procedia Engineering. 2015; 126: 446–450. 10.1115/1.4030668 PMC4462863 25994130

[20] Billiar KL, Sacks MS. Biaxial mechanical properties of the natural and glutaraldehyde treated aortic valve cusp–Part I: Experimental results. J Biomech Eng. 2000; 122: 23–30. 10.1115/1.429624 10790826

[21] Humphrey JD. Cardiovascular Solid Mechanics. New York: Springer-Verlag; 2002.

[22] Bovendeerd PHM, Arts T, Huyghe JM, van Campen DH, Reneman RS, Dependence of local left ventricular wall mechanics on myocardial fiber orientation: a model study, Journal of biomechanics. 1992; 25: 1129–1140. 1400513 10.1016/0021-9290(92)90069-d

[23] Sanchez-Quintana D, Anderson RH, Ho SY, Ventricular myoarchitecture in tetralogy of Fallot, Heart. 1996;17: 280–286. 10.1136/hrt.76.3.280 PMC484521 8868990

[24] Leonardi B, Taylor AM, Mansi T, Voigt I, Sermesant M, Pennec X, et, al Computational modelling of the right ventricle in repaired tetralogy of Fallot: can it provide insight into patient treatment? EUROPEAN HEART JOURNAL CARDIOVASCULAR IMAGING. 2013, 14(4):381–386. 10.1093/ehjci/jes239 23169758 PMC3597253

[25] Toussaint N, Mansi T, Delingette H, Ayache N, Sermesant M. An Integrated Platform for Dynamic Cardiac Simulation and Image Processing: Application to Personalised Tetralogy of Fallot Simulation, Proceedings of the Eurographics Workshop on Visual Computing for Biomedicine, VCBM 2008, Delft, The Netherlands, 2008.

[26] Geva T, Sandweiss BM, Gauvreau K, Lock JE, Powell AJ. Factors Associated With Impaired Clinical Status in Long-Term Survivors of Tetralogy of Fallot Repair Evaluated by Magnetic Resonance Imaging, Journal of the American College of Cardiology, 2004, 43(6):1068–1073. 10.1016/j.jacc.2003.10.045 15028368

[27] Lee C, Lee CH, Kwak JG, Kim SH, Shim WS, Lee SY, et, al Factors associated with right ventricular dilatation and dysfunction in patients with chronic pulmonary regurgitation after repair of tetralogy of Fallot: Analysis of magnetic resonance imaging data from 218 patients. The Journal of Thoracic And Cardiovascular Surgery, 2014, 148(6):2589–2595. 10.1016/j.jtcvs.2014.07.051 25173118

[28] Mansi T, Voigt I, Leonardi B, Pennec X, Durrleman S, Sermesant M, et, al A Statistical Model for Quantification and Prediction of Cardiac Remodelling: Application to Tetralogy of Fallot. IEEE Transactions on Medical Imaging, 2011, 30(9):1605–1616. 10.1109/TMI.2011.2135375 21880565

[29] Zhang H, Wahle A, Johnson RK, Scholz TD, Sonka M, 4-D Cardiac MR Image Analysis: Left and Right Ventricular Morphology and Function. IEEE TRANSACTIONS ON MEDICAL IMAGING, 2010, 29(2): 350–363. 10.1109/TMI.2009.2030799 19709962 PMC2849009

[30] del Nido PJ. Surgical management of right ventricular dysfunction late after repair of Tetralogy of Fallot: right ventricular remodeling surgery, Semin Thorac Cardiovasc Surg Pediatr Card Surg Annu. 2006; 29–34. 10.1053/j.pcsu.2006.02.007 16638544

[31] Geva T, Gauvreau K, Powell AJ, Cecchin F, Rhodes J, Geva J, et. al Randomized Trial of Pulmonary Valve Replacement With and Without Right Ventricular Remodeling Surgery, Circulation. 2010 9 14;122(11 Suppl):S201–8. 20837914 10.1161/CIRCULATIONAHA.110.951178 PMC2943672

[32] Zhou Z, Geva T, Rathod RH, Tang A, Yang C, Billiar KL, et, al Combining Smaller Patch, RV Remodeling and Tissue Regeneration in Pulmonary Valve Replacement Surgery Design May Lead to Better Post-Surgery RV Cardiac Function for Patients with Tetralogy of Fallot, MCB: Molecular & Cellular Biomechanics, 2018, 15(2):99–115.

[33] Ohayon J, Dubreuil O, Tracqui P, Le Floc'h S, Rioufol G, Chalabreysse L, et. al Influence of Residual Stress/Strain on the Biomechanical Stability of Vulnerable Coronary Plaques: Potential Impact for Evaluating the Risk of Plaque Rupture, Am. J. Physiol., 2007, 293(3):H1987–96. 10.1152/ajpheart.00018.2007 17604326

[34] Speelman L, Bosboom EM, Schurink GW, Buth J, Breeuwer M., Jacobs MJ, et. al, Initial Stress and Nonlinear Material Behavior in Patient-Specific AAA Wall Stress Analysis, J. Biomech., 2009,42(11):1713–9. 10.1016/j.jbiomech.2009.04.020 19447391

