# Supplementary material for: Ventricle stress/strain comparisons between Tetralogy of Fallot patients and healthy using models with different zero-load diastole and systole morphologies
Source: PLoS One. 2019 Aug 14;14(8):e0220328. doi: 10.1371/journal.pone.0220328 (PMC6693773; doi:10.1371/journal.pone.0220328)
Supplement: S1 Appendix — (DOCX) [file pone.0220328.s001.docx]

# S1 Appendix: Patient-specific myocardium material parameter value determination

In reality, RV/LV active contraction/relaxation lead to changes of sarcomere zero-stress length and myocardium material stiffness properties in a cardiac cycle. Sarcomere zero-stress length changes were approximated by our using of different systole and diastole zero-load geometries. Ventricle material parameters c_1_, c_2_, D_1_, D_2_, K_1_ and K_2_ in Equation (8) were numerically determined to match the CMR measured RV volume at each time step when MRI data were available (30 MRI time-points for each cardiac cycle). Since there was only one data (ventricle volume) to match, there was only one freedom to adjust to change the 6 parameters. We chose to keep c_2_, D_2_, and K_2_ values fixed and adjust c_1_, D_1_, and K_1_ proportionally until numerical volume matched MRI volume. An iterative method was used to determine the parameter values. Details are given below:

**Step 1:** An initial parameter values were set as c_1_=3.47 kPa, c_2_=0 kPa, D_1_=1.09 kPa, D_2_=3.0, K_1_=16.61 kPa, K_2_=3.0 based on our ex vivo biaxial data and prior experiences. They were called the current parameter values.

**Step 2 (time loop):** For time steps 1, 2, 30 (MRI data has 30 time steps), do Step 3-4 until final time step was reached.

**Step 3 (iteration loop):** For each time point, the RV/LV model was solved with the current material parameter values and pressure condition. Numerical RV volume was computed with Simpson’s method and slices from numerical solutions. The ratio of MRI-measured volume and numerical RV volume was computed. If the ratio was in the interval [0.998, 1.002] (0.2% error threshold), the iteration stopped and the current material parameters would be chosen as the results for this time step. We would then move forward to next time step and do Step 3 until the final time step was reached. . If the ratio was not in the interval [0.998, 1.002] (error > 0.2% threshold), proceed to Step 4.

**Step 4:** Let

$R=\frac{Numerical RV Volume}{MRI-measured RV Volume}$ (S-1)

be the ratio and multiply the current c_1_, D_1_, and K_1_ by R, update c_1_, D_1_, and K_1_ with their new values, go back to Step 3. Steps 3-4 were repeated until the final time step in a cardiac cycle was reached.

It should be noted that if R<1, the adjustments would make the materials softer so that the new numerical RV volume would increase. The opposite would happen for R>1.

The above procedure was programed and we were able to run the program semi-automatically for all the cases with occasional manual adjustments.
