# Supplementary material for: Ventricle stress/strain comparisons between Tetralogy of Fallot patients and healthy using models with different zero-load diastole and systole morphologies
Source: PLoS One. 2019 Aug 14;14(8):e0220328. doi: 10.1371/journal.pone.0220328 (PMC6693773; doi:10.1371/journal.pone.0220328)
Supplement: S1 Table — BE: Begin-Ejection; EE: End-Ejection; BF: Begin-Filling; EF: End-Filling. (DOCX) [file pone.0220328.s002.docx]

**S1 Table. Parameters in Mooney-Rivlin model for myocardium (c_2_=0 kPa, D_2_=3.0, K_2_=3.0). BE: Begin-Ejection; EE: End-Ejection; BF: Begin-Filling; EF: End-Filling**

| Groups | | Patients | Ejection Phase | | | | Filling Phase | | | |
| --- | --- | --- | --- | --- | --- | --- | --- | --- | --- | --- |
|  |  |  |  | c_1_(kPa) | D_1_(kPa) | K_1_(kPa) |  | c_1_(kPa) | D_1_(kPa) | K_1_(kPa) |
| Healthy Group | | P1 | BE | 4.17 | 1.31 | 19.94 | BF | 0.08 | 0.03 | 0.38 |
|  |  |  | EE | 0.07 | 0.02 | 0.33 | EF | 4.86 | 1.53 | 23.26 |
|  |  | P2 | BE | 4.51 | 1.42 | 21.60 | BF | 0.24 | 0.08 | 1.16 |
|  |  |  | EE | 0.21 | 0.07 | 1.00 | EF | 5.21 | 1.64 | 24.92 |
|  |  | P3 | BE | 3.12 | 0.98 | 14.95 | BF | 0.17 | 0.05 | 0.83 |
|  |  |  | EE | 0.14 | 0.04 | 0.66 | EF | 3.64 | 1.15 | 17.45 |
|  |  | P4 | BE | 4.86 | 1.53 | 23.26 | BF | 0.28 | 0.09 | 1.33 |
|  |  |  | EE | 0.26 | 0.08 | 1.25 | EF | 5.90 | 1.86 | 28.24 |
|  |  | P5 | BE | 4.86 | 1.53 | 23.26 | BF | 0.31 | 0.10 | 1.50 |
|  |  |  | EE | 0.28 | 0.09 | 1.33 | EF | 5.90 | 1.86 | 28.24 |
|  |  | P6 | BE | 3.47 | 1.09 | 16.61 | BF | 0.16 | 0.05 | 0.78 |
|  |  |  | EE | 0.16 | 0.05 | 0.75 | EF | 4.51 | 1.42 | 21.60 |
|  |  | HG Mean±SD | BE | 4.16 | 1.31 | 19.94 | BF | 0.21 | 0.06 | 1.00 |
|  |  |  |  | ±0.73 | ±0.23 | ±3.49 |  | ±0.09 | ±0.03 | ±0.40 |
|  |  |  | EE | 0.19 | 0.06 | 0.89 | EF | 5.00 | 1.58 | 23.95 |
|  |  |  |  | ±0.08 | ±0.02 | ±0.38 |  | ±0.87 | ±0.27 | ±4.15 |
| TOF Group | Better Outcome Group | P7 | BE | 6.25 | 1.97 | 29.91 | BF | 0.87 | 0.27 | 4.15 |
|  |  |  | EE | 0.80 | 0.25 | 3.82 | EF | 7.29 | 2.30 | 34.89 |
|  |  | P8 | BE | 8.68 | 2.73 | 41.54 | BF | 1.74 | 0.55 | 8.31 |
|  |  |  | EE | 1.63 | 0.51 | 7.81 | EF | 9.02 | 2.84 | 43.20 |
|  |  | P9 | BE | 6.94 | 2.19 | 33.23 | BF | 0.40 | 0.13 | 1.91 |
|  |  |  | EE | 0.35 | 0.11 | 1.66 | EF | 8.33 | 2.62 | 39.87 |
|  |  | P10 | BE | 3.12 | 0.98 | 14.95 | BF | 0.20 | 0.06 | 0.95 |
|  |  |  | EE | 0.19 | 0.06 | 0.90 | EF | 4.51 | 1.42 | 21.60 |
|  |  | P11 | BE | 2.78 | 0.87 | 13.29 | BF | 0.21 | 0.07 | 1.00 |
|  |  |  | EE | 0.17 | 0.05 | 0.83 | EF | 3.47 | 1.09 | 16.61 |
|  |  | P12 | BE | 2.95 | 0.93 | 14.12 | BF | 0.49 | 0.15 | 2.33 |
|  |  |  | EE | 0.38 | 0.12 | 1.83 | EF | 4.51 | 1.42 | 21.60 |
|  |  | BG Mean±SD | BE | 5.12 | 1.61 | 24.51 | BF | 0.65 | 0.21 | 3.11 |
|  |  |  |  | ±2.51 | ±0.79 | ±12.00 |  | ±0.58 | ±0.18 | ±2.80 |
|  |  |  | EE | 0.62 | 0.18 | 2.81 | EF | 6.19 | 1.95 | 29.63 |
|  |  |  |  | ±0.62 | ±0.18 | ±2.68 |  | ±2.32 | ±0.73 | ±11.09 |
|  | Worse Outcome Group | P13 | BE | 6.42 | 2.02 | 30.74 | BF | 0.19 | 0.06 | 0.91 |
|  |  |  | EE | 0.17 | 0.05 | 0.83 | EF | 8.68 | 2.73 | 41.54 |
|  |  | P14 | BE | 7.64 | 2.41 | 36.55 | BF | 1.32 | 0.42 | 6.31 |
|  |  |  | EE | 1.04 | 0.33 | 4.98 | EF | 10.41 | 3.28 | 49.84 |
|  |  | P15 | BE | 13.88 | 4.37 | 66.46 | BF | 3.99 | 1.26 | 19.11 |
|  |  |  | EE | 3.12 | 0.98 | 14.95 | EF | 14.58 | 4.59 | 69.78 |
|  |  | P16 | BE | 5.55 | 1.75 | 26.58 | BF | 0.30 | 0.09 | 1.41 |
|  |  |  | EE | 0.24 | 0.08 | 1.16 | EF | 7.29 | 2.30 | 34.89 |
|  |  | P17 | BE | 13.19 | 4.16 | 63.13 | BF | 2.43 | 0.77 | 11.63 |
|  |  |  | EE | 1.98 | 0.62 | 9.47 | EF | 14.23 | 4.48 | 68.12 |
|  |  | P18 | BE | 11.11 | 3.50 | 53.17 | BF | 2.92 | 0.92 | 13.96 |
|  |  |  | EE | 2.43 | 0.77 | 11.63 | EF | 12.84 | 4.05 | 61.47 |
|  |  | WG Mean±SD | BE | 9.63 | 3.03 | 46.10 | BF | 1.86 | 0.59 | 8.89 |
|  |  |  |  | ±3.57 | ±1.13 | ±17.10 |  | ±1.52 | ±0.48 | ±7.26 |
|  |  |  | EE | 1.50 | 0.47 | 7.17 | EF | 11.39 | 3.57 | 54.27 |
|  |  |  |  | ±1.20 | ±0.38 | ±2.78 |  | ±3.02 | ±0.95 | ±14.43 |
|  | TG Mean±SD | | BE | 7.38 | 2.32 | 35.30 | BF | 1.25 | 0.39 | 6.00 |
|  |  |  |  | ±3.77 | ±1.18 | ±18.04 |  | ±1.27 | ±0.40 | ±6.06 |
|  |  |  | EE | 1.04 | 0.33 | 4.9 | EF | 8.76 | 2.76 | 41.95 |
|  |  |  |  | ±1.02 | ±0.32 | ±4.86 |  | ±3.72 | ±1.17 | ±17.78 |
| All HG+TG Mean±SD | | | BE | 6.31 | 1.99 | 30.18 | BF | 0.90 | 0.29 | 4.33 |
|  |  |  |  | ±3.43 | ±1.08 | ±16.42 |  | ±1.14 | ±0.36 | ±5.45 |
|  |  |  | EE | 0.76 | 0.24 | 3.63 | EF | 7.51 | 2.37 | 35.95 |
|  |  |  |  | ±0.91 | ±0.29 | ±4.39 |  | ±3.53 | ±1.11 | ±16.90 |
